# Supplementary material for: microRNAs bidirectionally regulate FUT1 to modulate α-1,2-fucosylation and cancer-associated biology
Source: J Biol Chem. 2026 May 31;302(7):113217. doi: 10.1016/j.jbc.2026.113217 (PMC13320027; doi:10.1016/j.jbc.2026.113217)
Supplement: Supporting Figures and Table [file mmc4.docx]

**Supporting information on the main text, figures, and methods**

**Title**: microRNAs bidirectionally regulate FUT1 to modulate α-1,2-fucosylation and cancer-associated biology

**Authors:** Tigist Batu, Chu Thu, Lara K. Mahal*

**Affiliations:** ^1^Department of Chemistry, University of Alberta, Edmonton, Alberta, Canada T6G 2G2

* **Corresponding Author:** lkmahal@ualberta.ca

**This file contains:**

**Figures: S1-S8**

**Table: S1**

**Dataset: 1-3**


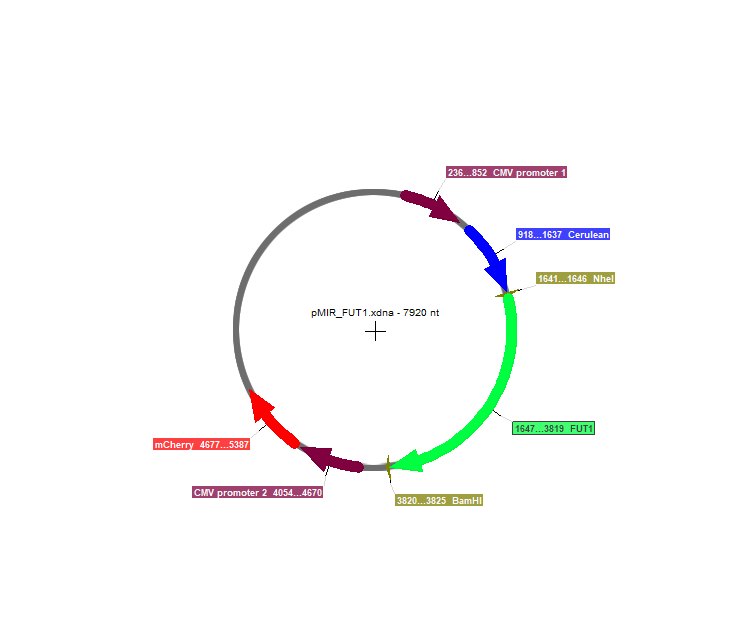


**Figure S1. pFmiR-FUT1 map**  pFmiR-FUT1 plasmid map. (Created using SnapGene).


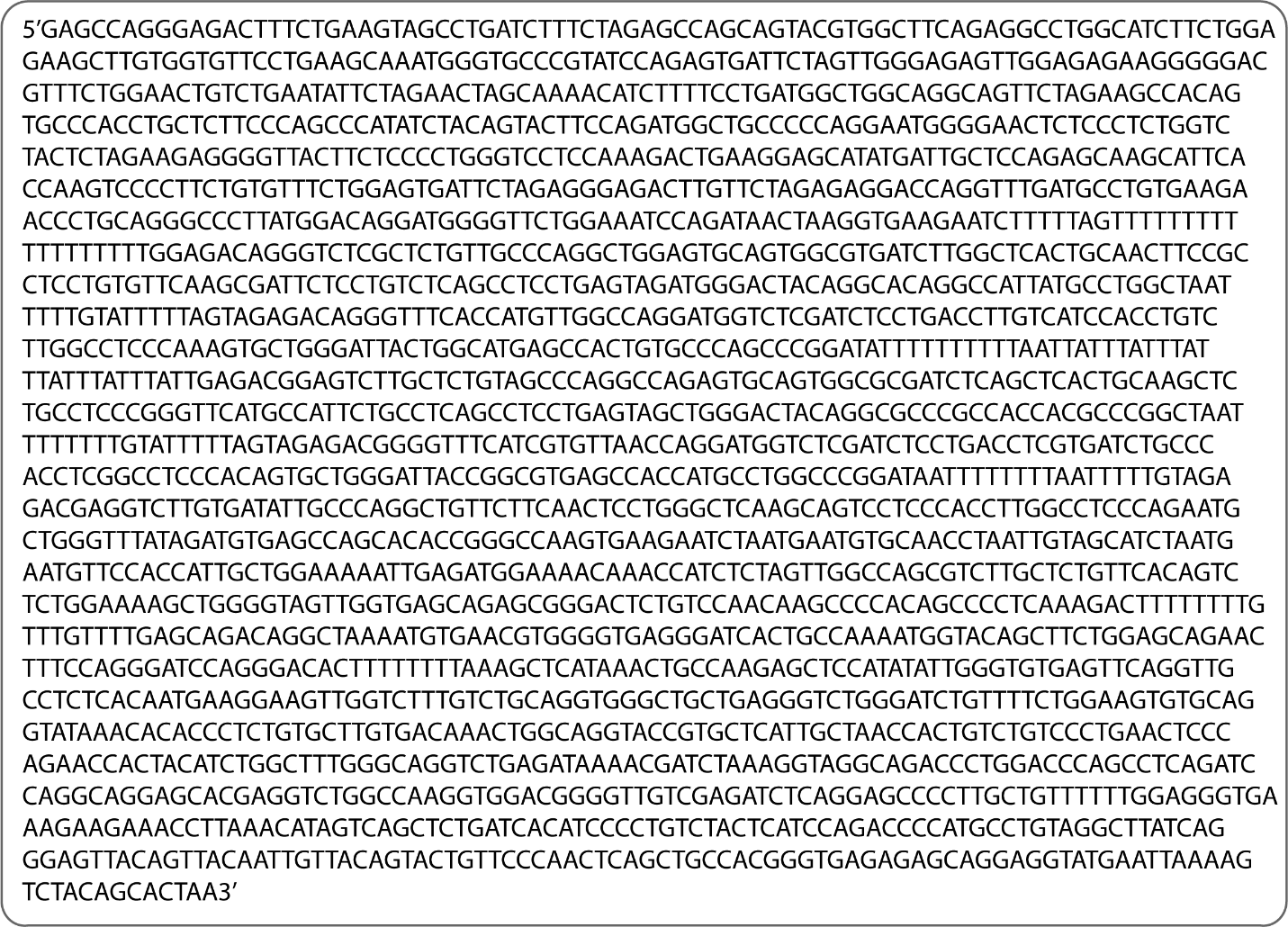


**Figure S2. FUT1 3’UTR sequence.** FUT1 3'UTR sequence (2173 base pairs).


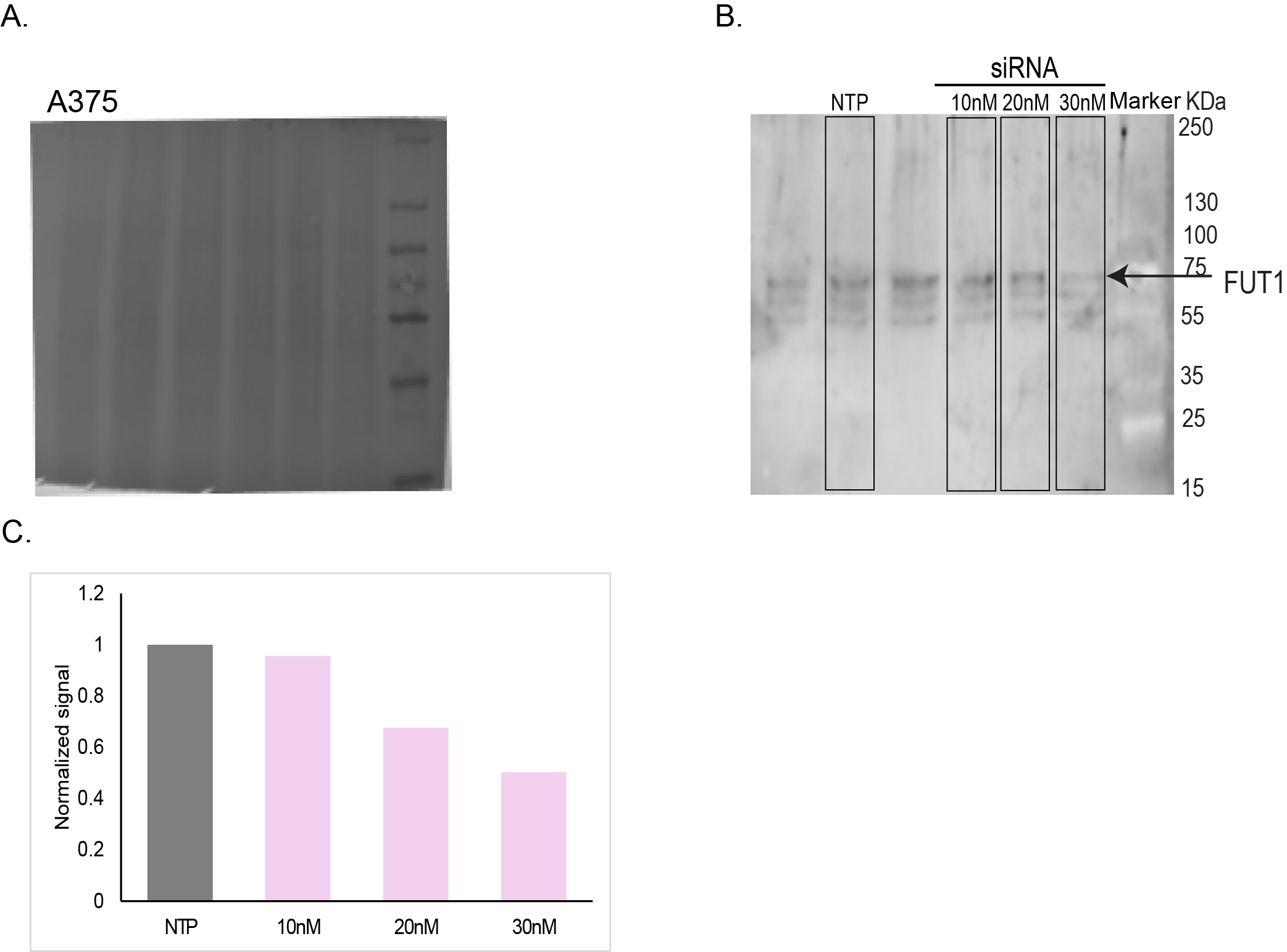


**Figure S3. Validation of ant-FUT1 antibody.**  siRNA against FUT1 was transfected into A375 using the standard protocol, NTP (30nM), siRNA(10,20,30nM). (A) Ponceau staining. (B) Whole western blot approx. Validated FUT1 band is at ~70 kDa. (C) Graphical representation of quantification of the top band using NIH Image. Note, the other bands did not lose intensity upon inhibition by siRNA


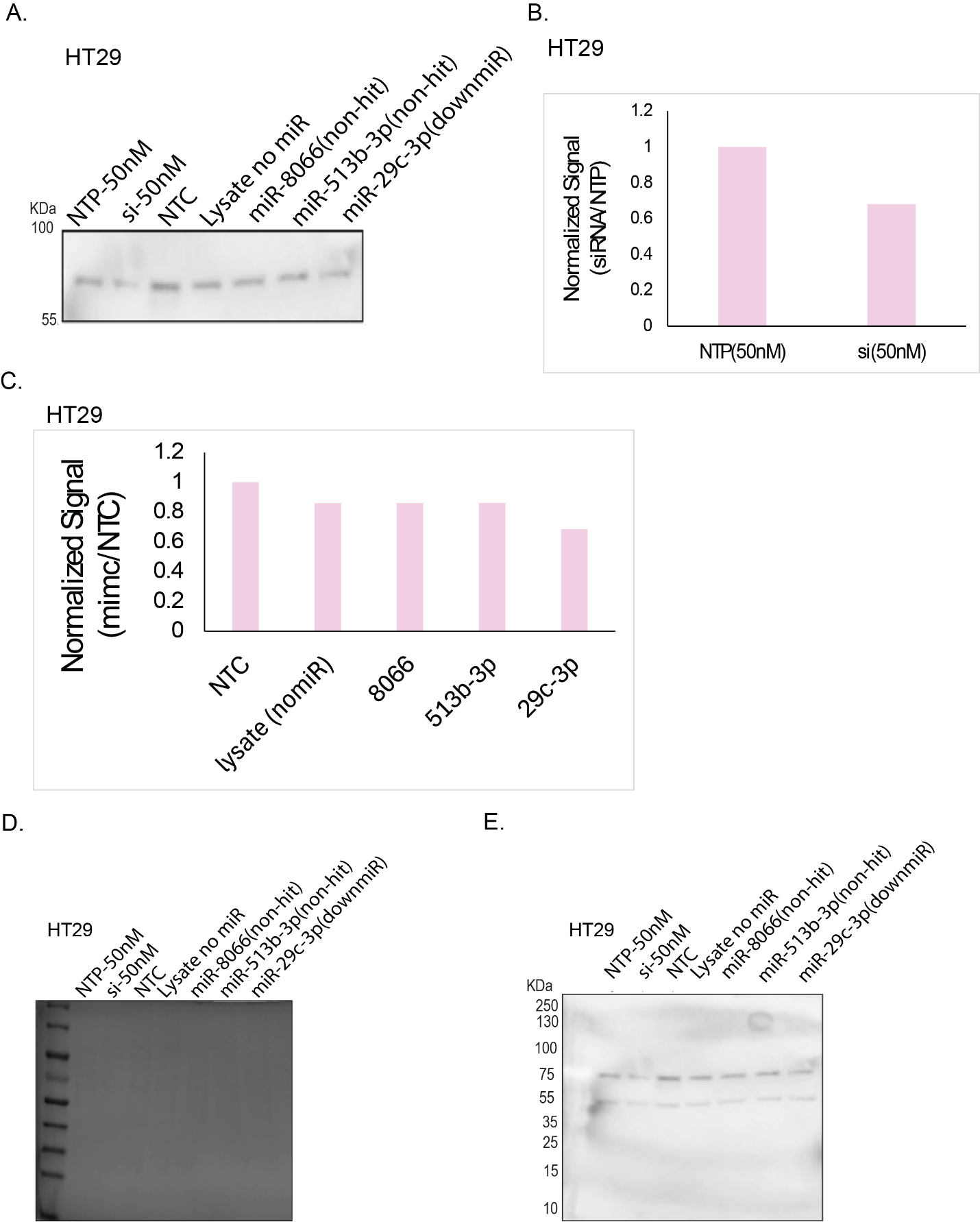


**Figure S4. Identification of a new non-targeting miRNA control (NTC*).** Candidate miRNAs from the median range of the miRFluR dataset were screened for their effect on FUT1 expression. (A) Western blot showing FUT1 levels following transfection with candidate miRNAs. (B) Quantification of normalized FUT1 signal comparing non-targeting pool (NTP) and siRNA controls. (C) Comparison of normalized FUT1 expression across tested candidates and miRNA controls. (D) Ponceau staining confirming equal loading. (E) Full western blot. miR-8066 produced a signal comparable to the lysate-only control, indicating no measurable effect on FUT1 expression, and was therefore selected as the non-targeting control (NTC*) for subsequent experiments.


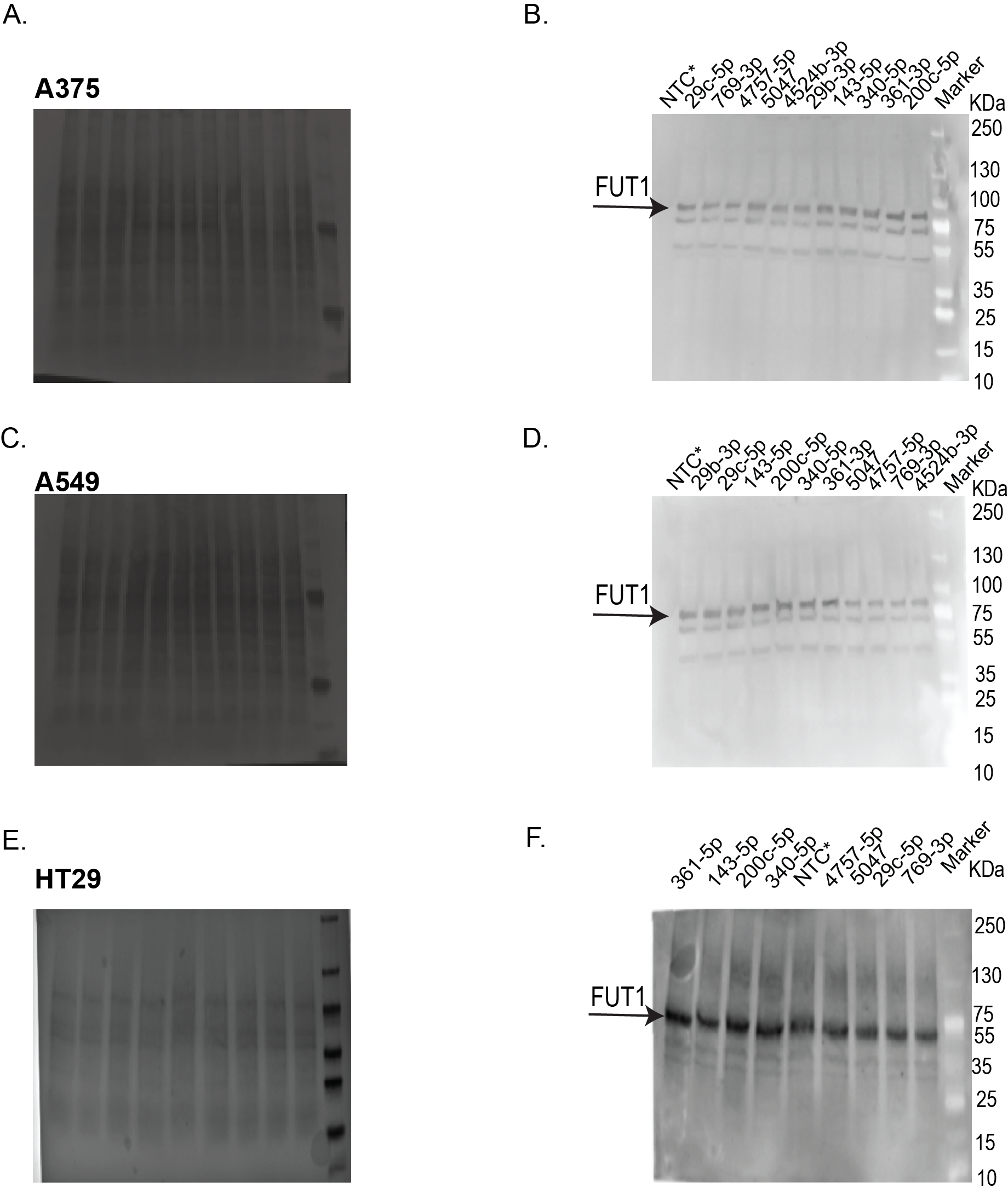


**Figure S5.** **Ponceau and whole Western blots for data shown in Fig. 3A.**  (A) Ponceau staining of blots used in **Fig. 3A** top (A375), (B) Whole Western blot for data shown in **Fig. 3B** (A375), (C) Ponceau staining of blots used in **Fig. 3A** middle (A549), (D) Whole Western blot for data shown in **Fig. 3C** (A549), (E) Ponceau staining of blots used in **Fig. 3A** bottom (HT29), (F) Whole Western blot for data shown in **Fig. 3D** (HT29). Note: the non-specific bands show cell line specific patterns.


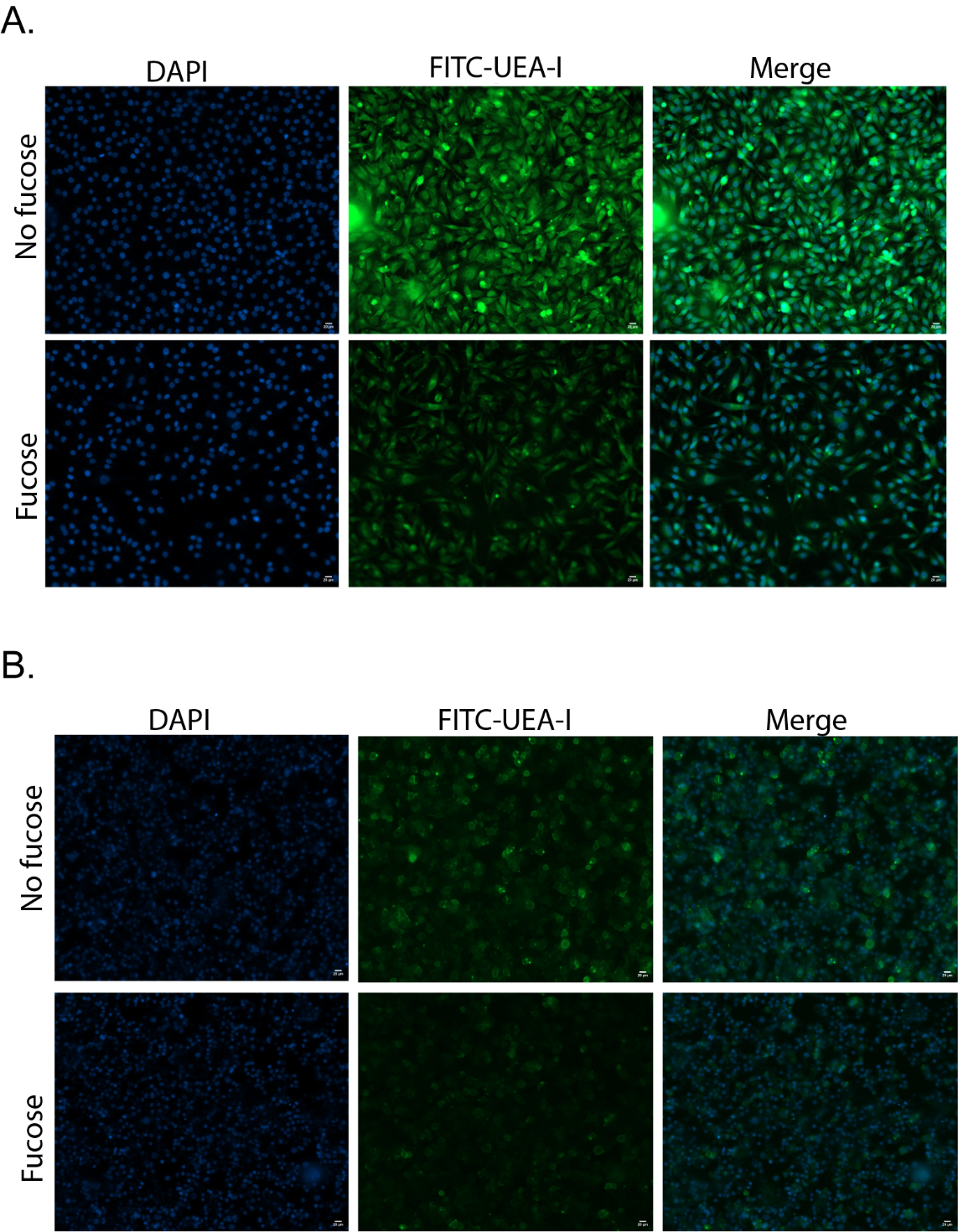


**Figure S6. Negative control for UEA-I binding lectin staining.** (A) A375, (B) HT29 To confirm the specificity of UEA-I lectin staining, a competitive inhibition control was performed using 100 mM free L-fucose. Cells were incubated with FITC-conjugated UEA-I in the presence or absence of L-fucose. L-fucose competes with α-1,2-fucosylated glycans for UEA-I binding and serves as a negative control for lectin specificity.


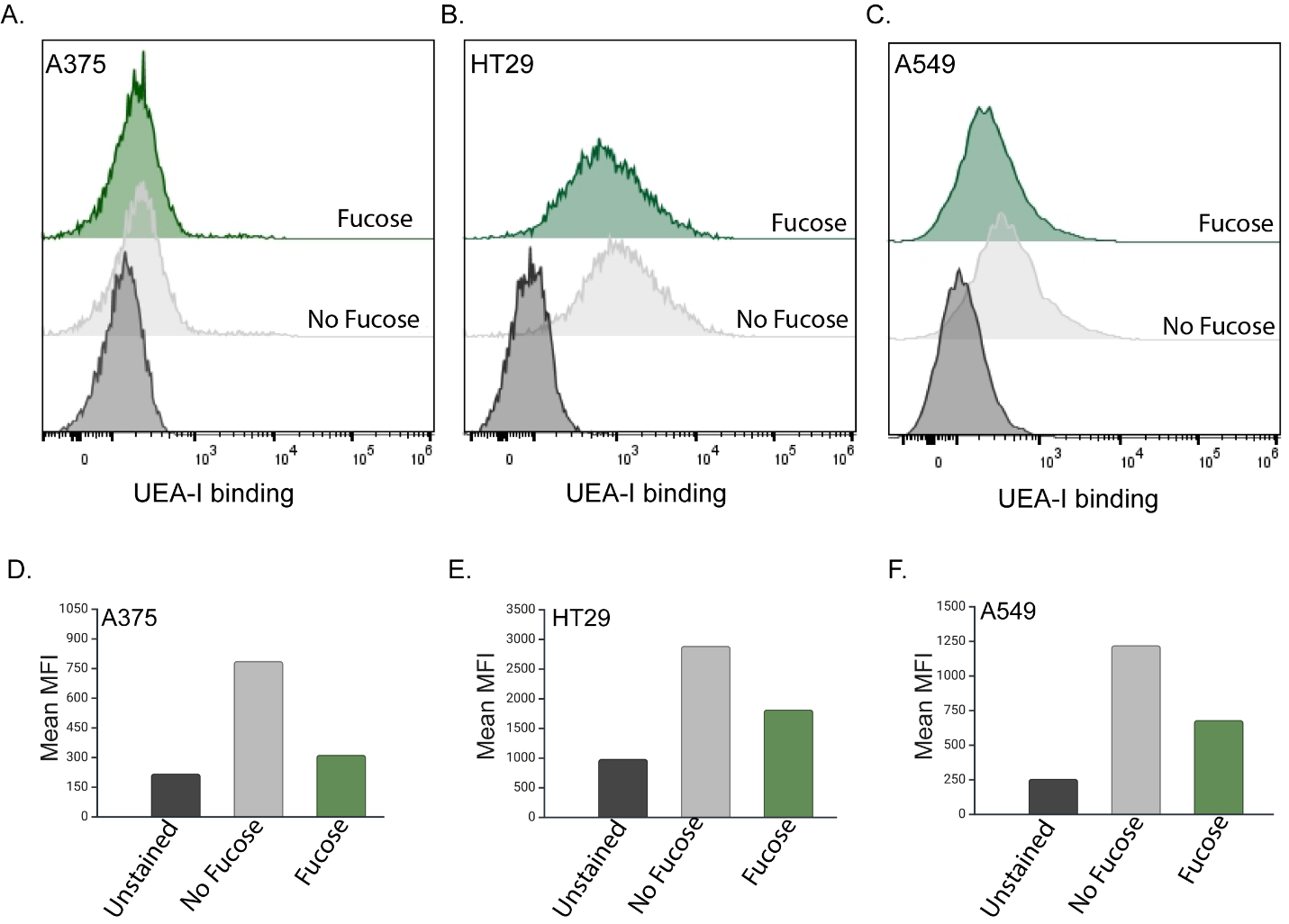


**Figure S7. Negative control for UEA-I binding flow cytometry.** (A) A375, (B) HT29

To validate the specificity of UEA-I binding in flow cytometry, a competitive inhibition assay was performed using free L-fucose. Cells were incubated with FITC-conjugated UEA-I in the presence or absence L-fucose. L-fucose competes with α-1,2-fucosylated glycans for UEA-I binding and therefore serves as a specificity control. After incubation, cells were washed with PBS and analyzed by flow cytometry. Mean fluorescence intensity (MFI) was calculated to quantify UEA-I binding.


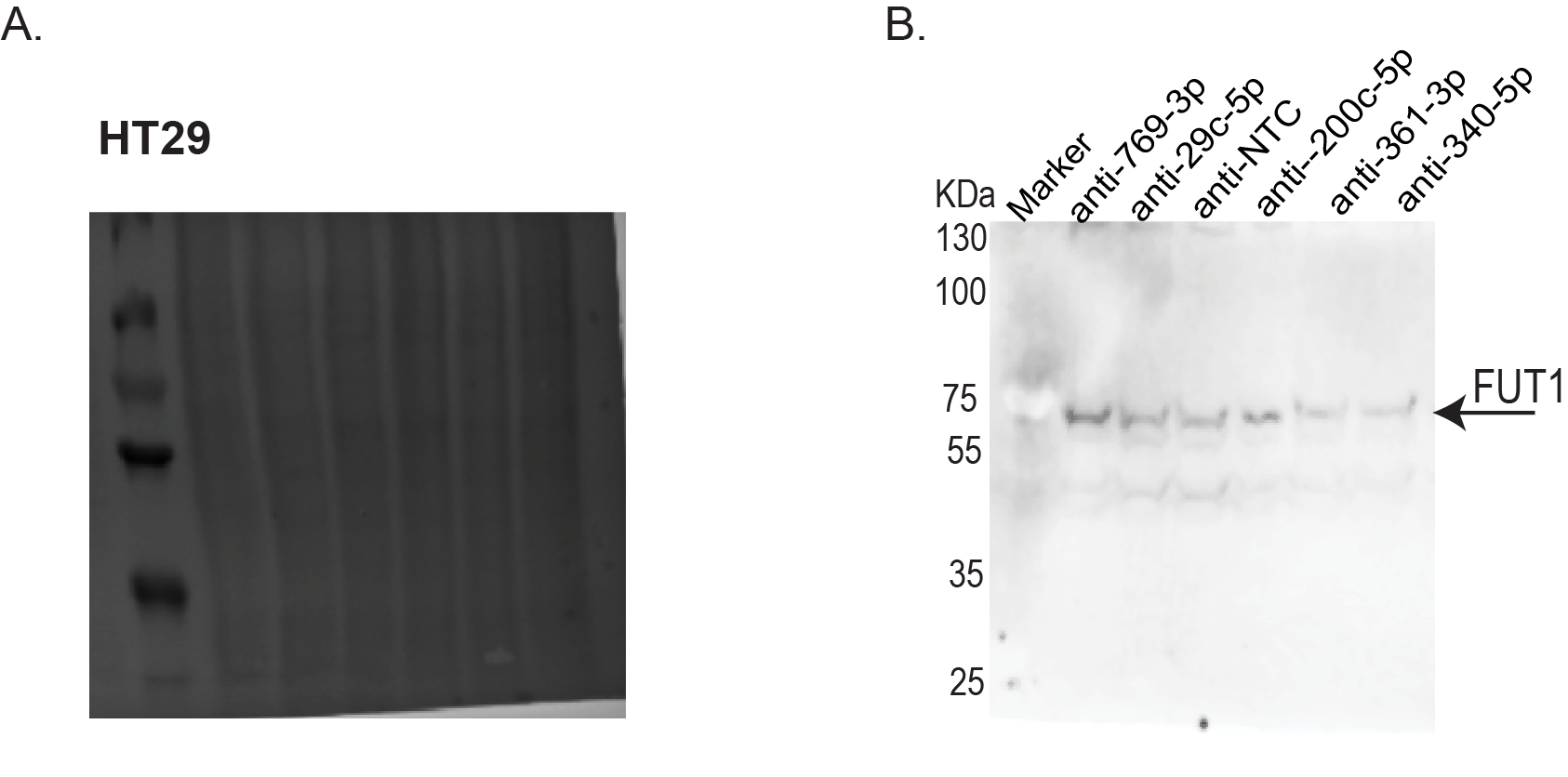


**Figure S8.** **Ponceau and whole Western blots for data shown in Fig. 5B.**  (A) Ponceau staining of blots used in **Fig. 5B** (HT29), (B) Whole Western blot for data shown in **Fig. 5B** (HT29).

**Table S1** Statistical significance for western blot experiments using one-sample *t-*test

| **miRs/anti-miRs** | **Cell line** | | |
| --- | --- | --- | --- |
|  | **A549** | **A375** | **HT29** |
|  | **One-sample *t*-test** | | |
| Down-miRs |  |  |  |
| 29c-5p | 0.0335 | 0.0119 | 0.0043 |
| 769-3p | 0.0127 | 0.0064 | 0.0609(NS) |
| 4757-5p | 0.0192 | 0.9701 (NS) | 0.2666(NS) |
| 5047 | 0.0761 (NS) | 0.0032 | 0.1466(NS) |
| 4524b-3p | 0.0815 (NS) | 0.0089 |  |
| Up-mirs |  |  |  |
| 29b-3p | 0.1487 (NS) | 0.0085 |  |
| 143-3p | 0.0355 | 0.0121 | 0.0002 |
| 340-5p | 0.0193 | 0.0015 | 0.0038 |
| 361-3p | 0.0064 | 0.0034 | 0.0008 |
| 200c-5p | 0.0384 | 0.0074 | 0.0257 |
| Down-miRs |  |  |  |
| anti-29c-3p |  |  | 0.0063 |
| anti-769-3p |  |  | 0.0075 |
| Up-mirs |  |  |  |
| anti-200c-5p |  |  | 0.3739(NS) |
| anti-340-5p |  |  | 0.0297 |
| anti-361-5p |  |  | 0.0119 |

**Dataset S1:** *Tab 1:* Total dataset, *Tab 2:* Dataset after removing plates with high error

*Tab 3:* QCed data (<15% error of measurement), *Tab 4:* Z-scored data.

**Dataset S2:** NCI-60 library miR-200c, FUT1, UEAI expression**.**

**Dataset S3:** miEAA enrichment analysis for FUT1 upmiRs (KEGG, MNDR).
